# Opuntiol Prevents Photoaging of Mouse Skin *via* Blocking Inflammatory Responses and Collagen Degradation

**DOI:** 10.1155/2020/5275178

**Published:** 2020-11-30

**Authors:** P. Veeramani kandan, Agilan Balupillai, G. Kanimozhi, Haseeb A. Khan, Abdullah S. Alhomida, Nagarajan Rajendra Prasad

**Affiliations:** ^1^Department of Biochemistry & Biotechnology, Annamalai University, Annamalainagar, 608 002 Tamil Nadu, India; ^2^Department of Biotechnology, Thiruvalluvar University, Serkadu, 632115 Vellore, Tamil Nadu, India; ^3^Department of Biochemistry, Dharmapuram Gnanambigai Government Arts College for Women, Mayiladuthurai, Tamil Nadu, India; ^4^Department of Biochemistry, College of Science, King Saud University, Riyadh 11451, Saudi Arabia

## Abstract

In the present study, we investigated the potential of opuntiol, isolated from *Opuntia ficus-indica*, against UVA radiation-mediated inflammation and skin photoaging in experimental animals. The skin-shaved experimental mouse was subjected to UVA exposure at the dosage of 10 J/cm^2^ per day for ten consecutive days (cumulative UVA dose: 100 J/cm^2^). Opuntiol (50 mg/kg b.wt.) was topically applied one hour before each UVA exposure. UVA (100 J/cm^2^) exposure induces epidermal hyperplasia and collagen disarrangement which leads to the photoaging-associated molecular changes in the mouse skin. Opuntiol pretreatment prevented UVA-linked clinical macroscopic skin lesions and histological changes in the mouse skin. Further, opuntiol prevents UVA-linked dermal collagen fiber loss in the mouse skin. Short-term UVA radiation (100 J/cm^2^) activates MAPKs through AP-1 and NF-*κ*B p65 transcriptional pathways and subsequently induces the expression of inflammatory proteins and matrix-degrading proteinases in the mouse skin. Interestingly, opuntiol pretreatment inhibited UVA-induced activation of iNOS, VEGF, TNF-*α*, and COX-2 proteins and consequent activation of MMP-2, MMP-9, and MMP-12 in the mouse skin. Moreover, opuntiol was found to prevent collagen I and III breakdown in UVA radiation-exposed mouse skin. Thus, opuntiol protects mouse skin from UVA radiation-associated photoaging responses through inhibiting inflammatory responses, MAPK activation, and degradation of matrix collagen molecules.

## 1. Introduction

The skin is the primary outmost layer of the human body, and it acts as an initial safeguard against harmful effects of environmental and biological factors [1]. The human skin essentially comprises three key sections, i.e., epidermis, dermis, and hypodermis. Additionally, the skin can be possessed with elastic and collagen fibers and proteoglycan-reliant matrices [2]. The environmental UV radiation belongs to the nonionizing part of electromagnetic radiation, which consists of three wavebands, i.e., UVA, UVB, and UVC [3]. Amongst them, UVA radiation (320–400 nm) has been considered as aging rays by induction of oxidative stress, mitogen-activated protein kinases (MAPKs), and inflammatory signaling [4]. The environmental UVA radiation-linked photoaging is characterized by degradation of collagen, premature skin aging, wrinkle formation, and erythema of the skin [5]. UVA radiation induces excessive production of reactive oxygen species (ROS) through cellular photosensitizers that lead to the signatures of photoaging and inflammation in the dermal skin layers [6].

UVA exposure creates a large quantity of ROS that can induce stress-activated MAPK signaling [7]. The UVA-linked MAPK stimulations generally activate nuclear factor kappa B (NF-*κ*B) and activator protein-1 (AP-1) translocation, which resulted in the overexpression of several biomarkers that are associated with inflammation and photoaging [8, 9]. Matrix metalloproteinases (MMPs) are crucial endopeptidases which are involved in the remodelling and degradation of collagen and extracellular matrix in the skin [10]. Collagen is an essential main building block of the human skin, and 90% of collagen is present in the total skin. Collagen provides strengthening of the skin, and degradation of collagen during the UVA exposure resulted in the wrinkling of the skin, a sign of premature photoaging [11]. The actions of MMP-1, MMP-9, and MMP-12 have been reported to be involved in UVA-induced photoaging. The UVA-associated MMP-1expression critically participates in the degradation of type I collagen [12]. MMP-9, also called gelatinase, degrades type I and IV collagen in the extracellular matrix (ECM) [13].

UVA radiation induces inflammatory reactions that are directly associated with the pathogenesis of photoaging [14]. The NF-*κ*B and AP-1 translocation from cytosol to the nucleus has an important event to regulate proinflammatory mediators upon UVA exposure that could provoke vascular endothelial growth factor (VEGF), cyclooxygenase-2 (COX-2), and inducible nitric oxide synthase (iNOS) which hasten the pathogenesis of photoaging [8, 15, 16]. Moreover, tumor necrosis factor-*α* (TNF-*α*) and interleukins are critically involved in NF-*κ*B-dependent inflammatory reaction during UVA radiation-associated photoaging [17]. Thus, inhibition of NF-*κ*B and AP-1 transcriptional activity is considered a pivotal strategy for the prevention of UVA radiation-associated inflammation and photoaging [18].

The natural phytochemicals from a variety of plant sources have magnificently attracted researchers for developing new phytomedicine products against UVA radiation-mediated photoaging. Cactus *Opuntia ficus-indica* is a family of *Cactaceae* highly present in dry and desert regions of the Earth. The prickly pad possesses several antioxidant nutrients [19] [20],. As *Opuntia ficus-indica* receives high levels of UV ambience, it might have developed defences against the detrimental effects of UV light as exemplified by the presence of several flavonol glycosides that possess strong UV-absorbing properties [21, 22]. We recently isolated opuntiol from *Opuntia ficus-indica* and reported its protective effect against UVA-induced oxidative changes in 3T3 skin cells [23, 24]. In this study, we report the preventive effect of opuntiol against UVA radiation-associated photoaging responses in the mouse skin.

## 2. Materials and Methods

### 2.1. Reagents and Antibodies

Monoclonal antibodies such as cyclooxygenase-2 (COX-2), nuclear factor kappa B p65 (NF-*κ*B p65), vascular endothelial growth factor (VEGF), tumor necrotic factor-alpha (TNF-*α*), interleukin-6 (IL-6), inducible nitric oxide synthase (iNOS), and anti-mouse and goat anti-mouse IgG-HRP polyclonal antibodies were acquired from Santa Cruz, USA. All other chemicals, solvents, and other analytical grade chemicals were obtained from SD Fine Chemicals, Mumbai, India.

### 2.2. Animals and UVA Irradiation

Male Swiss albino mice (8 weeks old) were purchased from Charles Biogen from Bangalore and housed at the Annamalai University Animal Husbandry. The Institutional Animal Ethical Committee (IAEC) approved the study protocol. The mice were acclimatized for at least 7 days ahead of the experimentation in an aerated and temperature-regulated room and have proper access to water *ad libitum*. The mice were randomized into four groups, and each group consists of 10 mice. Group 1 served as untreated control mice; group 2 mice served as opuntiol (50 mg/kg b.wt.) alone; group 3 mice were irradiated with UVA alone (100 J/cm^2^) and acted as negative control; group 4 mice received opuntiol (50 mg/kg b.wt.) 1 h before UVA radiation. The mouse skin hair was completely removed using hair removal cream three days ahead of experimental initiation. The dorsal skin was irradiated to UVA (365 nm), and the dose rate was given as 10 J/cm^2^ per day for ten days; totally, the mice received 100 J/cm^2^ [25]. After the treatment period, the mice were subjected to anaesthesia (60 mg/kg ketamine) and the mice were sacrificed as per the guidelines given by IAEC. The dorsal portions of the skin were used for various analyses.

### 2.3. Examination of Clinical Severity of Skin Injury

The clinical severity appearance in the skin lesions was evaluated macroscopically in mouse skin [26]. The severity of the skin injury was tested four times a week by at least three independent dermatologists. The formation of skin disorders like dryness, erythema, hemorrhage, skin redness, and erosion was scaled and scored as 0 (none), 1 (mild), 2 (moderate), and 3 (severe). The sum of the individual scores was considered as the dermatitis score.

### 2.4. Pathological Studies

After the end of photoaging treatment, skin biopsy was collected by the addition of 10% formaldehyde and the skin sections were examined for histological changes using hematoxylin and eosin (H&E) staining, Masson's trichrome staining, and Verhoeff van Gieson staining.

After completion of treatment, the experimental mouse skin tissue was homogenised by a homogenizer. Then, collagen I and collagen III contents were determined using commercial kits and the protocol was followed as per the manufacturer's instruction (BioVision Incorporated, USA).

### 2.5. Immunohistochemistry

After the end of photoaging experiments, the mouse skin sections were prepared and treated with 5% goat serum. Appropriate primary antibodies (1 : 100 dilution) were added to the skin sections and incubated overnight. Then, the secondary antibody was added (1 : 200 dilution of biotinylated anti-mouse IgG) and stained with hematoxylin. The skin specimens were examined with a light microscope (20x). Dermatologists confirmed all the changes in pathological expression [25].

### 2.6. Western Blotting

The dorsal skin was collected and was treated with 2.5 U/mL Dispase® II (neutral protease, grade II) in PBS solution overnight at 4°C. After the incubation time, the epidermis and dermis layers were gently separated using a scalpel. The dermis layer was stored at −80°C before use. The proteins were extracted from the dermal layer using RIPA buffer that is consist of protease cocktail inhibitor (1 *μ*g/1 *μ*L). The cytosolic and nuclear extracts were prepared by the method previously described [27]. The amounts of protein concentration were measured by using the NanoDrop spectrophotometer (Thermo Scientific, Austria). Briefly, 50 *μ*g of protein samples from each group was fractionated on 8-10% SDS-PAGE gel, and it was transferred to a nitrocellulose membrane (Bio-Rad, Germany) using a semidry apparatus (Bio-Rad). The membrane was blocked with 5% nonfat milk (blocking solution) for 1 h at 4°C; then, respective primary antibody (1 : 1000 in 5% BSA solution) was added and incubated overnight at 4°C. The membranes were washed with TBST buffer for 15 min intervals and then incubated with horseradish peroxidase-conjugated secondary antibody (1 : 5000 in nonfat milk) and kept for incubation for 1 h at 37°C. After the TBST buffer washing, the protein bands were spotted using a chemiluminescence detection method and the images were quantified by using Image Studio software (LI-COR).

### 2.7. Statistical Analysis

The statistical analysis was carried out by one-way analysis of variance (ANOVA) using SPSS (Statistical Package for the Social Sciences). The group means were compared to the statistical tool Duncan's multiple range test (DMRT). The results were considered statistically significant if the *P* value is <0.05.

## 3. Results

### 3.1. Acute Toxicity of Opuntiol in Swiss Albino Mice

The administration of different concentrations of opuntiol (5-50 mg/kg b.wt.) has not induced any mortality during the 14-day observation period (Supplementary data 1). However, 16.6% of animals died when the opuntiol dose was raised to 100 mg/kg b.wt. A further increase in the dose of opuntiol (200 mg/kg b.wt.) resulted in 33.3% mortality. About 50% reduction in the survival of mice was observed at 400 mg/kg b.wt. Further, 66.6% of the mice died when the drug dose was increased to 800 mg/kg b.wt. Finally, 1600 mg/kg b.wt. of opuntiol induced 83.3% mortality. Opuntiol did not show toxicity and mortality up to 50 mg/kg b.wt., and hence, we selected this nontoxic concentration for photoprotection studies.

### 3.2. Opuntiol Suppresses UVA-Mediated Skin Injury

**Figure 1:**
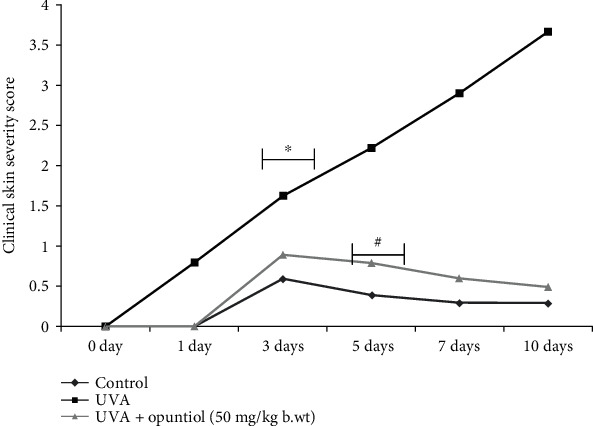
Effect of opuntiol (50 mg/kg b.wt.) on UVA radiation-mediated clinical macroscopic skin lesions. The severity of the UVA (100 J/cm^2^) irradiation-mediated skin damages was tested four times a week. The skin disorders like dryness, erythema, hemorrhage, skin redness, and erosion were scored as 0 (none), 1 (mild), 2 (moderate), and 3 (severe). The data represent means ± SD (*n* = 6), and values not sharing a common marking (∗ and #) differ significantly at *P* < 0.05 (DMRT).

The role of opuntiol on UVA-mediated dorsal skin section injuries was assessed by several clinical parameters such as erythema, dryness, edema, and erosion. The clinical skin severity score was magnificently increased during the UVA (100 J/cm^2^) exposure compared to the untreated control mouse. In contrast, opuntiol (50 mg/kg b.wt.)-treated mice showed significant attenuation in the severity scores at 3 days after UVA radiation, and the ameliorative effect was persistent until 10 days following the exposure (Figure 1).

### 3.3. Opuntiol Prevents UVA Radiation-Induced Histopathological Changes and Hyperplasia

**Figure 2:**
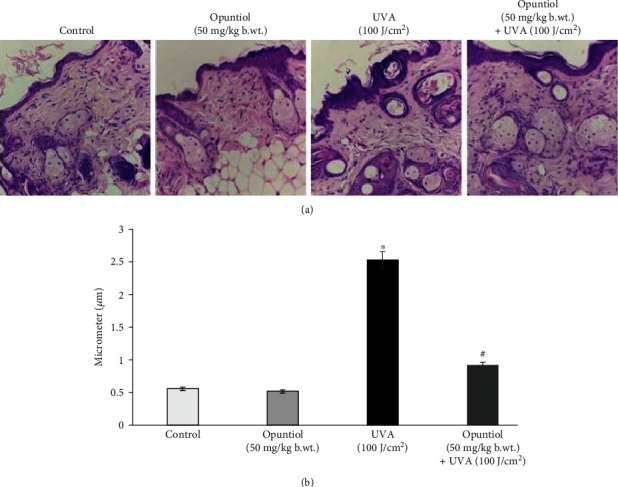
Effect of opuntiol on UVA-induced epidermal hyperplasia and histological changes in the mouse skin. (a) Opuntiol (50 mg/kg b.wt.) on UVA (100 J/cm^2^)-mediated histological changes in mouse skin. After the treatment, skin sections are stained with H&E (20x magnification). (b) Bar diagram represents epidermal hyperplasia and epidermal thickness which was measured by Magnus Pro software; a minimum of six animals and six experiments for each specimen were taken. The data represent means ± SD (*n* = 6), and values not sharing a common marking (∗ and #) differ significantly at *P* < 0.05 (DMRT).

The dorsal portion of the skin damage and skin epidermal hyperplasia was analyzed by hematoxylin and eosin staining. The thickness of the skin epidermis was measured at 25 different sites from each section, and the mean value of epidermal thickness was measured. Significant hyperplasia was observed in the UVA (100 J/cm^2^)-exposed region of the epidermis. Conversely, opuntiol (50 mg/kg b.wt.) pretreatment prevented the UVA radiation-associated epidermal hyperplasia in the mouse skin (Figure 2).

### 3.4. Opuntiol Prevents UVA-Linked Dermal Collagen Fiber Loss and Dermal Collagen Density

**Figure 3:**
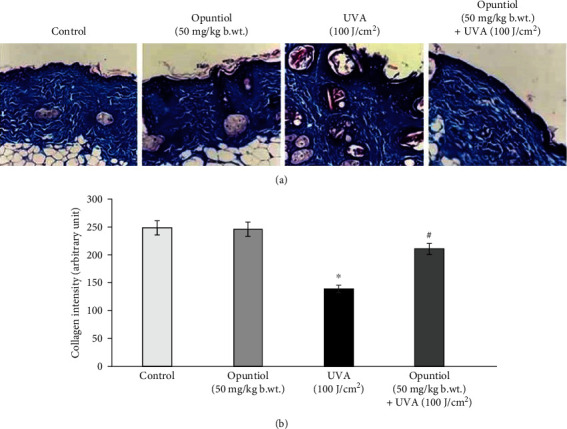
Opuntiol prevents UVA-linked dermal collagen fiber loss in mouse skin. Degradation of dermal collagen fibers was determined in samples dissected after the treatment with UVA (100 J/cm^2^) radiation and/or opuntiol (50 mg/kg b.wt.), stained with Masson's trichrome. Representative images (20x) show control, opuntiol, UVA radiation, and opuntiol plus UVA radiation. The bar diagram represents collagen density which was measured by Magnus Pro software. The data represent means ± SD (*n* = 6), and values not sharing a common marking (∗ and #) differ significantly at *P* < 0.05 (DMRT).

The preventive role of opuntiol on the UVA radiation-associated dermal collagen fiber loss was analyzed by Masson trichrome staining. Control and opuntiol (50 mg/kg b.wt.) alone-treated mouse skin sections show collagen fibers stained with blue dye; and it was abundantly observed in the dermis layer of the dorsal skin. However, there was an apparent decrease of blue staining in the UVA (100 J/cm^2^)-irradiated mouse dermis. It indicates the destruction of collagen fibers in the dermis during UVA exposure. Conversely, opuntiol (50 mg/kg b.wt.) pretreatment decreased the collagen fiber degradation, which was evident by increased blue staining in opuntiol plus UVA-irradiated mouse skin (Figure 3).

**Figure 4:**
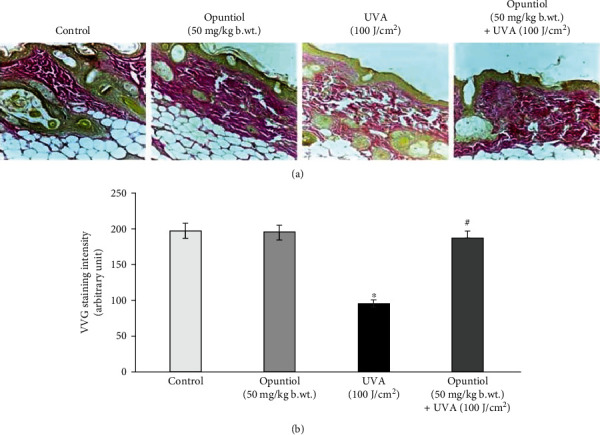
Opuntiol prevents UVA-linked dermal collagen density in mouse skin. Degradation of collagen was determined in samples dissected after the treatment with UVA (100 J/cm^2^) radiation and/or opuntiol (50 mg/kg b.wt.), stained with Verhoeff van Gieson staining. Representative images (20x) show control, opuntiol, UVA radiation, and opuntiol with UVA radiation. The bar diagram represents collagen density which was measured by Magnus Pro software. The data represent means ± SD (*n* = 6), and values not sharing a common marking (∗ and #) differ significantly at *P* < 0.05 (DMRT).

Furthermore, opuntiol on UVA-mediated collagen density was analyzed by Verhoeff van Gieson staining in the skin section. UVA radiation (100 J/cm^2^) appreciably reduced dermal collagen density in the mouse skin. In contrast, the topical pretreatment of opuntiol (50 mg/kg b.wt.) prevented the amount of collagen density in UVA-irradiated mouse skin (Figure 4).

### 3.5. Opuntiol Prevents MAPK, NF-*κ*B, and AP-1 Expressions in UVA-Irradiated Mouse Skin

**Figure 5:**
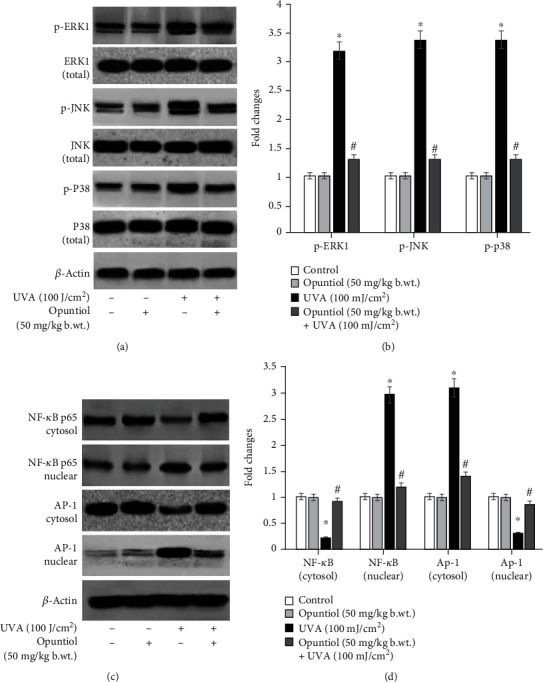
Opuntiol prevents MAPK, NF-*κ*B, and AP-1 expressions in UVA (100 J/cm^2^)-irradiated mouse skin. (a) Western blot analysis of opuntiol (50 mg/kg b.wt.) on UVA-linked tp-ERK1, p-JNK, and p-p38 and its total protein expression in mouse skin. (b) The bar diagram represents the quantification of protein which was measured by Image Studio software (LI-COR). The phosphorylated form of MAPK proteins was normalized by total protein of MAPKs, and *β*-actin was used to ensure the equal volume of protein loaded. (c) Western blot analysis of opuntiol on UVA-linked translocation of NF-*κ*B and AP-1 expression in mouse skin. The translocation of NF-*κ*B and AP-1 expression was analyzed by cytosolic and nuclear extract of protein samples. (d) The bar diagram represents the quantification of proteins which was analyzed by Image Studio software (LI-COR). *β*-Actin was used to normalize the examined protein and to ensure the equal volume of protein loaded The data represent means ± SD from three independent experiments, and values not sharing a common marking (∗ and #) differ significantly at *P* < 0.05 (Duncan's multiple range test (DMRT)).

Opuntiol on UVA-exposed expression of inflammatory MAPKs and transcriptional factors like NF-*κ*B and AP-1 was assessed by western blot analysis. UVA irradiation (100 J/cm^2^) induces a significant increment of phosphorylated ERK-1, JNK, and p38 expression when compared to nonirradiated mouse skin. The topical pretreatment of opuntiol (50 mg/kg b.wt.) extremely prevented UVA-induced overexpression of phosphorylated ERK-1, JNK, and p38 expression in the mouse skin. Moreover, UVA (100 J/cm^2^) enhanced the translocation of NF-*κ*B and AP-1 into the nucleus from the cytosol. We noticed increased NF-*κ*B and AP-1 expression in nuclear extract and decreased NF-*κ*B and AP-1 expression in the cytosolic fraction. Conversely, opuntiol pretreatment prevented UVA irradiation-induced translocation of NF-*κ*B AP-1 into the nucleus in the mouse skin cells (Figure 5).

### 3.6. Opuntiol Prevents Inflammatory Protein Expressions in UVA-Irradiated Mouse Skin

**Figure 6:**
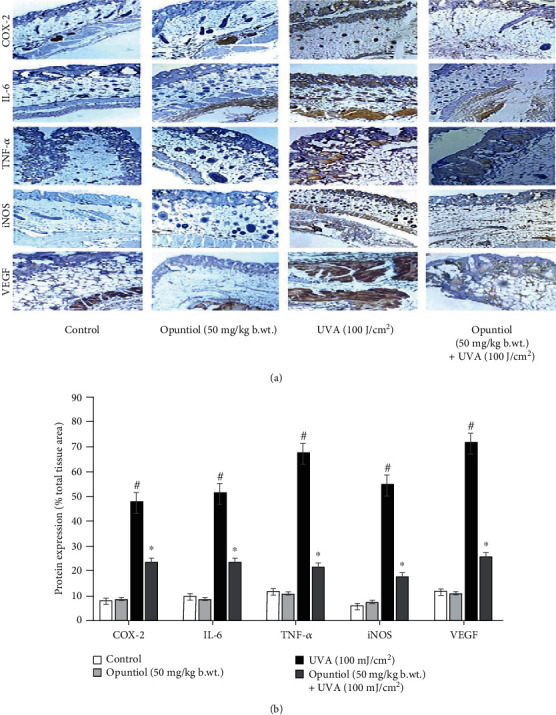
Effect of opuntiol on UVA-induced inflammatory protein expression in mouse skin. (a) The expression pattern of IL-6, TNF-*α*, COX-2, iNOS, and VEGF was analyzed by immunohistochemistry. Twenty-four hours following the last UVA irradiation, the mouse was sacrificed; the dorsal skin was used for immunohistochemistry. Representative photomicrographs (20x) illustrate IL-6, TNF-*α*, COX-2, iNOS, and VEGF expression in the mouse skin. (b) Densitometry analysis of IL-6, TNF-*α*, COX-2, iNOS, and VEGF expression in UVA and/or opuntiol-treated mouse skin. The histogram data was measured by Magnus Pro software, and the data were presented as a percentage of total tissue area. The data represent means ± SD from three independent experiments, and values not sharing a common marking (∗ and #) differ significantly at *P* < 0.05 (DMRT).

Opuntiol on UVA-exposed expression of inflammatory proteins such as IL-6, TNF-*α*, COX-2, iNOS, and VEGF was assessed by immunohistochemistry analysis. UVA (100 J/cm^2^)-irradiated mouse skin sections clearly show increased expression of IL-6, TNF-*α*, COX-2, iNOS, and VEGF evidenced by higher brown color staining in the skin sections. However, opuntiol (50 mg/kg b.wt.) topical treatment prevented the UVA-mediated expression of IL-6, TNF-*α*, COX-2, iNOS, and VEGF proteins in the mouse skin (Figure 6).

### 3.7. Opuntiol Prevents MMP, Collagen I, and Collagen III Expression in UVA-Irradiated Mouse Skin

**Figure 7:**
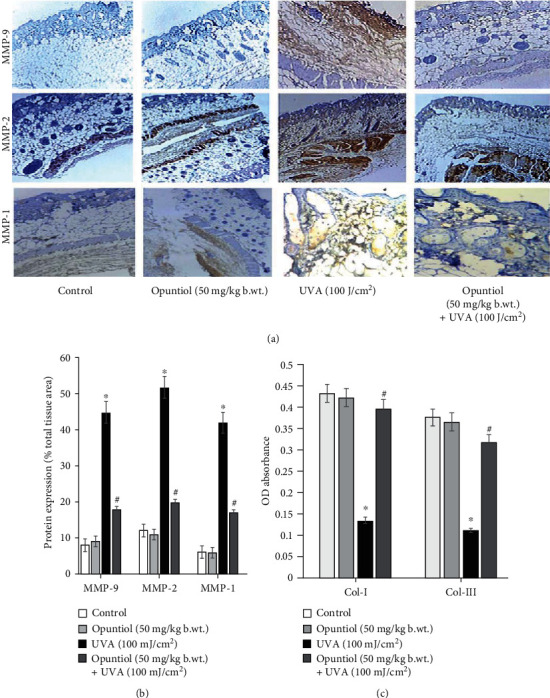
Opuntiol prevents MMP, collagen I, and collagen III expression in UVA (100 J/cm^2^)-irradiated mouse skin. (a) Effect of opuntiol (50 mg/kg b.wt.) on UVA-linked expression of MMP-9, MMP-2, and MMP-1 expression was analyzed by immunohistochemistry. Twenty-four hours following the last UVA irradiation, the mouse dorsal skin was collected for immunohistochemistry. Representative photomicrographs (20x) illustrate MMP-2 and MMP-9 expression. (b) Densitometry analysis of MMP-9, MMP-2, and MMP-1 expression in UVA radiation and/or opuntiol-treated mouse skin. The histogram data was measured by Magnus Pro software, and the data were presented as a percentage of total tissue area. (c) Effect of opuntiol on UVA-linked collagen I and collagen III expression in mouse skin. The content of collagen I and collagen III was determined using commercial kits. The data represent means ± SD from three independent experiments, and values not sharing a common marking (∗ and #) differ significantly at *P* < 0.05 (DMRT).

Mouse skin sections were used for the analysis of MMP-1, MMP-2, and MMP-9 protein expression. We observed higher brown spots in UVA (100 J/cm^2^)-exposed mouse skin as a measure of MMP-1, MMP-2, and MMP-9 overexpression. Opuntiol (50 mg/kg b.wt.) treatment before UVA radiation diminished brown dots in the mouse skin section that showed reduced expression of MMP-1, MMP-2, and MMP-9. Furthermore, opuntiol on UVA-mediated collagen degradation was analyzed. The study showed that UVA (100 J/cm^2^) radiation diminishes collagen I and collagen III in the mouse skin. In contrast, opuntiol pretreatment prevents UVA-induced depletion of collagen I and collagen III in the mouse skin. These results indicate that opuntiol significantly prevents the MMPs and degradation of collagen during UVA exposure in the mouse skin (Figure 7).

## 4. Discussion

Skin photodamages are marked as skin thickening, diminution in skin elasticity, and formation of wrinkles. Wrinkles are an apparent sign of cutaneous aging, and it reflects the sun-exposed areas [28]. We observed marked clinically oriented skin severity score in short-term UVA-exposed mouse skin which is tightly associated with erythema, dryness, edema, and erosion (Figure 1). The antioxidant plant-derived phytochemicals are reported to prevent UVA-mediated wrinkle formation [29]. We found that initially the topical treatment of opuntiol ahead of UVA radiation drastically decreased the severity scores at 3 days after UVA radiation and the preventive effect was continual until 10 days after the exposure. These results suggest that opuntiol has a protective effect against UVA-mediated skin injury.

The UVA radiation-mediated photoaging and skin injury have been characterized by decreased elasticity of the skin, wrinkle formation, and heavy damages of the extracellular matrix [30]. Furthermore, skin inflammation and keratin thickening also contribute to the signs of photoaging [31]. Histopathological examination reveals that exposure to short-term UVA light caused photodamages in the mouse skin which was evident by the altered skin architecture. The photodamaged mouse skin showed the sign of solar elastosis with altered collagen arrangements. We observed that opuntiol pretreatment prevents UVA radiation-mediated alterations in skin elasticity and epidermal hyperplasia. These results were closely associated with the results of Masson trichrome staining, which confirms the collagen density. Masson trichrome staining and VVG staining confirmed that opuntiol protects the loss of collagen density in UVA-exposed mouse skin. The previous result indicated that rambutan peel phenolics appreciably inhibited the UV-induced skin structure modification and protected the dermal collagen fibers in the mouse model [32].

Numerous studies have noted that UVA radiation induces a hallmark of photoaging events such as activation of MAPKs in the skin cells [33, 34]. The exposure to UVA heavily accelerates ROS-mediated phosphorylation of JNK, ERK1, and p38 which resulted in AP-1 and NF-*κ*B transactivation. Activation of UVA radiation-mediated AP-1 and NF-*κ*B expression was tightly associated with the array of inflammatory and photoaging signaling. Interestingly, we found that opuntiol prevents p-JNK, p-ERK1, p-p38, AP-1, and NF-*κ*B expression in UVA-exposed mouse skin. Previously, *Opuntia humifusa* fruit extract ameliorates UVA radiation-induced expression of MAPKs in mouse models [35]. Further, UVA radiation-mediated MAPK signaling activates NF-*κ*B and AP-1 to liberate inflammatory cytokines and mediators such as COX-2, TNF-*α*, VEGF, iNOS, and IL-6 [36]. Overexpression of these mediators induces inflammatory responses in the mouse skin during UVA exposure. In this present work, opuntiol inhibited inflammatory responses by downregulating the expression of COX-2, TNF-*α*, VEGF, iNOS, and IL-6 in UVA-exposed mouse skin. We previously reported that *α*-pinene, a naturally occurring phytochemical, suppresses UVA-induced inflammatory mediators such as COX-2, TNF-*α*, VEGF, iNOS, and IL-6 in the mouse skin^25^. Moreover, it has been reported that *Opuntia humifusa* inhibits UVB-induced carcinogenesis by reducing inflammatory response elements like COX-2, iNOS, and proinflammatory cytokines in the hairless mouse model [37]. This supporting information suggests that opuntiol may potentially prevent UVA-induced inflammation in the mouse skin.

Numerous investigations have showed that UV radiation induces inflammation in the skin which resulted in both intrinsic and extrinsic aging [32, 38]. The photoaging process has specifically been triggered by several proinflammatory mediators such as prostaglandin E2 (PGE2), COX-2, iNOS, TNF-*α*, IL-1*β*, and IL-6 receptors [39]. Upon UVA irradiation, these inflammatory mediators damage fibroblast cells through overexpression of MMPs [39]. Matrix metalloproteinases (MMPs) are the foremost collagenolytic enzymes accountable for skin collagen degradation and depletion of the extracellular matrix [40]. MMP-1 specifically possesses collagenase activity whereas MMP-2 and MMP-9 are responsible for gelatinase activity [41]. It has been reported that activation of MMPs requires AP-1 and NF-*κ*B transactivation to the nucleus during UVA exposure [40]. Western blot results show that UVA irradiation significantly upregulates AP-1- and NF-*κ*B-mediated overexpression of MMP-1, MMP-2, and MMP-9 in the mouse skin. Conversely, opuntiol pretreatment significantly prevents the depletion of MMP-1, MMP-2, and MMP-9 in UVA-irradiated mouse skin. Several flavonoids inhibit UVA-induced MAPK-mediated MMP expression in human dermal fibroblasts [42, 43]. This study reveals that opuntiol, a novel flavonoid, also bears the capability of inhibitory role against UVA-mediated MMPs. Repetitive exposure to UVA causes amendment in the skin composition [36]. Collagen has been considered as a critical component in the skin that performs function of the skin, especially maintaining extracellular matrix and skin elasticity. The depletion of collagen is a major phenomenon of photoaging [44]. Collagen I and collagen III are crucial biomarkers associated with the elasticity of the skin. There are numerous studies which reported the downregulation of collagen I and collagen III in the pathogenesis of photoaging [45, 46]. In the present work, opuntiol considerably enhanced the skin architecture by the augmented level of collagen I and collagen III in UVA-irradiated mouse skin. Tea polyphenols from *Ilex kudingcha* inhibit UVB-induced skin aging via enhancing the expression of collagen I and collagen III and TIMP-1 in SKH1 hairless mice [47]. Recently, we reported the antioxidant and photoprotective effect of opuntiol against UVA radiation-induced oxidative damages [24]. Therefore, the antioxidant potential of opuntiol might be attributed for its protection against short-term UVA-induced inflammatory and photoaging responses in the mouse skin.

## 5. Conclusion

We conclude that opuntiol prevents UVA-associated adverse effects such as inflammation and photoaging via suppression of MAPK signaling and prevention of nuclear translocation of NF-*κ*B and AP-1 in the mouse skin cells. Further, opuntiol prevents activation of matrix degrading MMPs in the mouse skin dermal layers. Moreover, opuntiol protects mouse skin by preventing depletion of collagen fibers during exposure to UVA radiation. Therefore, opuntiol may be possibly used as a prospective natural antiphotoaging agent. However, further investigations are needed to study its sunscreen potential when compared to already existing synthetic commercial antiphotoaging agents before its cosmetic applications.

## Data Availability

Data are available within the article or its supplementary materials.

## Abbreviations

UV: Ultraviolet radiation
UVA: Ultraviolet A radiation
MAPKs: Mitogen-activated protein kinases
AP-1: Activator protein-1
NF-*κ*B: Nuclear factor kappa B
VEGF: Vascular endothelial growth factor
TNF-*α*: Tumor necrosis factor-*α*
COX-2: Cyclooxygenase-2
MMPs: Matrix metalloproteinases
MMP-2: Matrix metalloproteinase-2
MMP-9: Matrix metalloproteinase-9
MMP-12: Matrix metalloproteinase-12
ECM: Extracellular matrix
ROS: Reactive oxygen species
iNOS: Inducible nitric oxide synthase
NF-*κ*B p65: Nuclear factor kappa B p65
IL-6: Interleukin-6
H&E staining: Hematoxylin and eosin staining
ANOVA: Analysis of variance
SPSS: Statistical Package for the Social Sciences
DMRT: Duncan's multiple range test.

## Supplementary Materials

Supplementary Materials S.1: dose selection study: acute toxicity of opuntiol in Swiss albino mice. Opuntiol did not show toxicity and mortality up to 50 mg/kg b.wt., and hence, we selected this nontoxic concentration for photoprotection studies.
