# EXPRESSION OF CONCERN: Opuntiol Prevents Photoaging of Mouse Skin *via* Blocking Inflammatory Responses and Collagen Degradation

**DOI:** 10.1155/2024/9806462

**Published:** 2024-10-08

**Authors:** Oxidative Medicine and Cellular Longevity

**EXPRESSION OF CONCERN:** P. V. Kandan, A. Balupillai, G. Kanimozhi, H. A. Khan, A. S. Alhomida, and N. R. Prasad, “Opuntiol Prevents Photoaging of Mouse Skin *via* Blocking Inflammatory Responses and Collagen Degradation,” *Oxidative Medicine and Cellular Longevity*, 2020, no. 1 (2020): e5275178, https://doi.org/10.1155/2020/5275178.

This Expression of Concern for [1], which was published online on 30 November 2020 in Wiley Online Library (https://onlinelibrary.wiley.com/), has been published by agreement between the journal Editor-in-Chief, Jeannette Vasquez-Vivar; and John Wiley & Sons Ltd. The Expression of Concern has been agreed due to overlaps observed between the Opuntiol/VEGF panel presented in Figure 6(a) and the UVA/MMP2 panel shown in Figure 7(a). Further overlaps were also observed between the Control/VEGF panel presented in Figure 6(a) and the Opuntiol/MMP-2 panel presented in Figure 7(a). The authors explained that the errors were a result of data mismanagement. Although the authors were able to provide some raw data, the editors have lost confidence in the data presented and would like to issue an Expression of Concern to alert the readers.
