# Supplementary material for: Opuntiol Prevents Photoaging of Mouse Skin via Blocking Inflammatory Responses and Collagen Degradation
Source: Oxid Med Cell Longev. 2020 Nov 30;2020:5275178. doi: 10.1155/2020/5275178 (PMC7721505; doi:10.1155/2020/5275178)
Supplement: Supplementary Materials — S.1: dose selection study: acute toxicity of opuntiol in Swiss albino mice. Opuntiol did not show toxicity and mortality up to 50 mg/kg b.wt., and hence, we selected this nontoxic concentration for photoprotection studies. [file 5275178.f1.docx]

| **Mortality on different day post drug treatment** | | | | | | | | | | | | | | | | |
| --- | --- | --- | --- | --- | --- | --- | --- | --- | --- | --- | --- | --- | --- | --- | --- | --- |
| **Opuntiol (mg/kg b.wt.)** | **1** | **2** | **3** | **4** | **5** | **6** | **7** | **8** | **9** | **10** | **11** | **12** | **13** | **14** | **% Mortality** | **Survivors/ Total** |
| 5 |  |  |  |  |  |  |  |  |  |  |  |  |  |  | 0 | 6/6 |
| 10 |  |  |  |  |  |  |  |  |  |  |  |  |  |  | 0 | 6/6 |
| 20 |  |  |  |  |  |  |  |  |  |  |  |  |  |  | 0 | 6/6 |
| 50 |  |  |  |  |  |  |  |  |  |  |  |  |  |  | 0 | 6/6 |
| 100 |  |  |  |  |  |  |  |  |  |  |  |  |  | 1 | 16.6 | 1/6 |
| 200 |  |  |  |  |  |  |  |  |  |  |  | 1 | 1 |  | 33.3 | 2/6 |
| 400 |  |  |  |  |  |  |  | 1 |  |  | 1 | 1 |  |  | 50 | 3/6 |
| 800 |  |  |  |  |  |  |  |  |  |  |  | 1 | 1 | 2 | 66.6 | 5/6 |
| 1600 |  |  |  |  |  |  |  |  |  | 2 | 2 | 1 |  |  | 83.3 | 6/6 |

**S.1****. Dose Selection Study: Acute toxicity of opuntiol in Swiss albino mice**

Opuntiol did not showed toxicity and mortality up to 50 mg/kg b,wt and hence we selected this non-toxic concentration for photoprotection studies.
